# Preliminary study on optimizing head position for endovascular treatment of anterior communicating artery aneurysms

**DOI:** 10.3389/fnins.2026.1936580

**Published:** 2026-08-26

**Authors:** Jin-Rong Zhang, Jing-Yu Li, Shu-Gai Liu, Zheng-Jun Wu, Qiu-Xia Liang, Xiao-Hong Yin, Bo Tan, Qiao-Jun Peng, Xiao-Ming Wang

**Affiliations:** 1 Neurological Medicine Center, Guangyuan Central Hospital Affiliated to North Sichuan Medical College, Guangyuan, Sichuan, China; 2 Department of Infectious Diseases, Guangyuan Central Hospital Affiliated to North Sichuan Medical College, Guangyuan, Sichuan, China; 3 Department of Neurology, Affiliated Hospital of North Sichuan Medical College, Nanchong, Sichuan, China

**Keywords:** anterior communicating artery aneurysm, endovascular therapy, functional outcome, head position, randomized controlled trial

## Abstract

**Objective:**

To evaluate the effects of optimized head positioning on procedural efficiency, device utilization, safety, and short-term postoperative activities of daily living in patients undergoing endovascular treatment for anterior communicating artery aneurysms (ACoAA), compared with conventional head positioning, and thereby assess its clinical value.

**Methods:**

In this single-center randomized controlled study, 86 patients with ACoAAs scheduled for endovascular treatment at Guangyuan Central Hospital, Affiliated to North Sichuan Medical College, between September 2022 and September 2025 were randomly assigned to a conventional-position group or an optimized-position group. One patient in the conventional group did not undergo the procedure; therefore, 85 patients were included in the final analysis (conventional group, *n* = 42; optimized group, *n* = 43). Outcomes included total procedure time, stent-delivery catheter placement time, embolization catheter placement time, coil embolization time, number of coils used, stent use, perioperative complications, and short-term neurological and functional outcomes.

**Results:**

The final analysis included 85 patients. The baseline data of the two groups were comparable. Compared with the conventional group, the optimized group had shorter total procedure times for both stent-assisted coil embolization and standalone coil embolization, shorter catheter-placement and coil-embolization times, and a lower stent-use rate (44.2% vs. 66.7%, *p* = 0.037). No statistically significant between-group differences were observed in the number of coils used, the perioperative complication rate, postoperative modified Rankin Scale (mRS) scores, or changes in mRS. Several other short-term neurological and functional measures favored the optimized group.

**Conclusion:**

Optimized head positioning was associated with shorter procedure times and lower stent use, without an evident increase in perioperative complications. Several short-term neurological and functional measures also favored the optimized position. This strategy is simple and readily implementable, supporting its potential for broader clinical application.

## 1 Introduction

Anterior communicating artery aneurysm (ACoAA) account for approximately 30–35% of intracranial aneurysms and have a substantial risk of rupture (approximately 40%) (Pietrantonio et al., 2020; Swiatek et al., 2024). As the parent artery of ACoAA, the anterior communicating artery (ACoA) converges the A1 and A2 segments of bilateral anterior cerebral arteries, its own main blood vessels, bilateral recurrent artery of Heubner (RAH), and numerous perforating vessels, collectively nourishing important tissues such as the deep basal ganglia, medial temporal lobe, and optic chiasm. It is the only core vascular hub in the skull base that simultaneously nourishes the deep nuclei and cortex (Chen et al., 2020). Due to the structural characteristics and anatomical complexity of ACoAA, the treatment of ACoAA requires consideration of both the eradication of aneurysms and the protection of surrounding blood vessels and perforating branches. At present, definitive treatment primarily includes microsurgical clipping (MC) and endovascular therapy (EVT). EVT has become the first-line treatment for intracranial aneurysms (IA) due to its advantages of lower incidence of complications and less trauma (Van Donkelaar et al., 2020; Müller et al., 2025; Schwab et al., 2025a). Conventional EVT techniques include standalone coil embolization (SCE) and stent-assisted coil embolization (SACE).

Management of ACoAA remains technically challenging with both MC and EVT (Lee et al., 2025). Previous studies have attempted to improve the success rate of IA surgery and reduce the incidence of complications by adopting appropriate head positions (Ivan et al., 2019; Yang et al., 2024). Ozdemir et al. (2014) quantified optimal viewing angles for ACoAA using vascular cast models from different orientations and proposed position-dependent surgical strategies for aneurysm exposure (Hackett et al., 2023). Similarly, Orakdogen et al. (2024) performed a meta-analysis and proposed a three-dimensional projection-based classification system of the neurovascular anatomy; however, their findings were primarily applicable to microsurgical planning rather than endovascular procedures. However, these studies remain limited as a guide for optimal head positioning in EVT. In particular, their applicability for achieving optimal visualization of ACoAA during endovascular procedures has not been validated. Schwab et al. (2025b) further investigated whether a universal optimal head position exists in EVT to improve aneurysm visualization, but the research results ultimately demonstrated heterogeneity in optimized positions among different IA, and did not find the optimal position for full exposure of ACoAA.

In the conventional head position for EVT of ACoAAs, the patient is supine with the craniocaudal axis parallel to the imaging table, and an anteroposterior (AP) projection is used initially. A Towne projection (TP) can be obtained by adjusting the craniocaudal angulation of the C-arm to improve visualization of the skull base and reduce overlap between bony structures and vascular images. However, this conventional position has significant spatial limitations. The presence of the operating table and the patient’s body restrict C-arm motion in three-dimensional space, and excessive angulation may increase the risk of gantry–table or gantry–patient collision. Although the three-dimensional roadmapping (3D-RM) technology provides multiple excellent angle options, it is limited by the mechanical constraints of the C-arm system, and some ideal working angles selected in 3D reconstruction cannot be reproduced in actual operation, which may result in suboptimal visualization of the working projection for ACoAA. The full display of spatial structures such as the aneurysm body, aneurysm neck, and surrounding blood vessels is essential for the success of EVT (Cieściński et al., 2012). Therefore, an optimized head position that enables comprehensive and stable visualization of ACoAA urgently needs to be explored.

We therefore conducted a single-center, prospective, randomized controlled trial involving 85 patients undergoing EVT for ACoAA. We hypothesized that 15° posterior head extension from 90° to 105° would expand the effective sagittal projection range, improve the available working projection, and facilitate procedural efficiency without compromising perioperative safety. The effects of the optimized and conventional head positions on procedure time, device utilization, complications, and short-term postoperative neurological and functional outcomes were compared.

## 2 Patients and methods

### 2.1 Participants

The study was approved by the Ethics Committee of Guangyuan Central Hospital Affiliated to North Sichuan Medical College (approval no. GYZXLLK2025018). We used a composite operating room equipped with a medical angiography X-ray system (Azurion 7M20; Philips Medical Systems Nederland B.V.). Consecutive patients with ACoAAs who were scheduled to undergo EVT between September 2022 and September 2025 were screened. The inclusion criteria were as follows: (1) ACoAA confirmed by computed tomography angiography or digital subtraction angiography; (2) planned endovascular embolization using SCE or SACE; (3) willingness to comply with follow-up; and (4) provision of written informed consent. The exclusion criteria were as follows: (1) a craniocervical junction deformity that prevented the required head positioning; (2) additional aneurysms requiring simultaneous treatment; (3) previous treatment of the ACoAA with MC or EVT; (4) a severe coagulation disorder or a contraindication to contrast media; (5) inability to tolerate the procedure because of a critical or life-threatening condition; and (6) inability or unwillingness to complete follow-up and reassessment.

### 2.2 Study procedures

**Figure 1:**
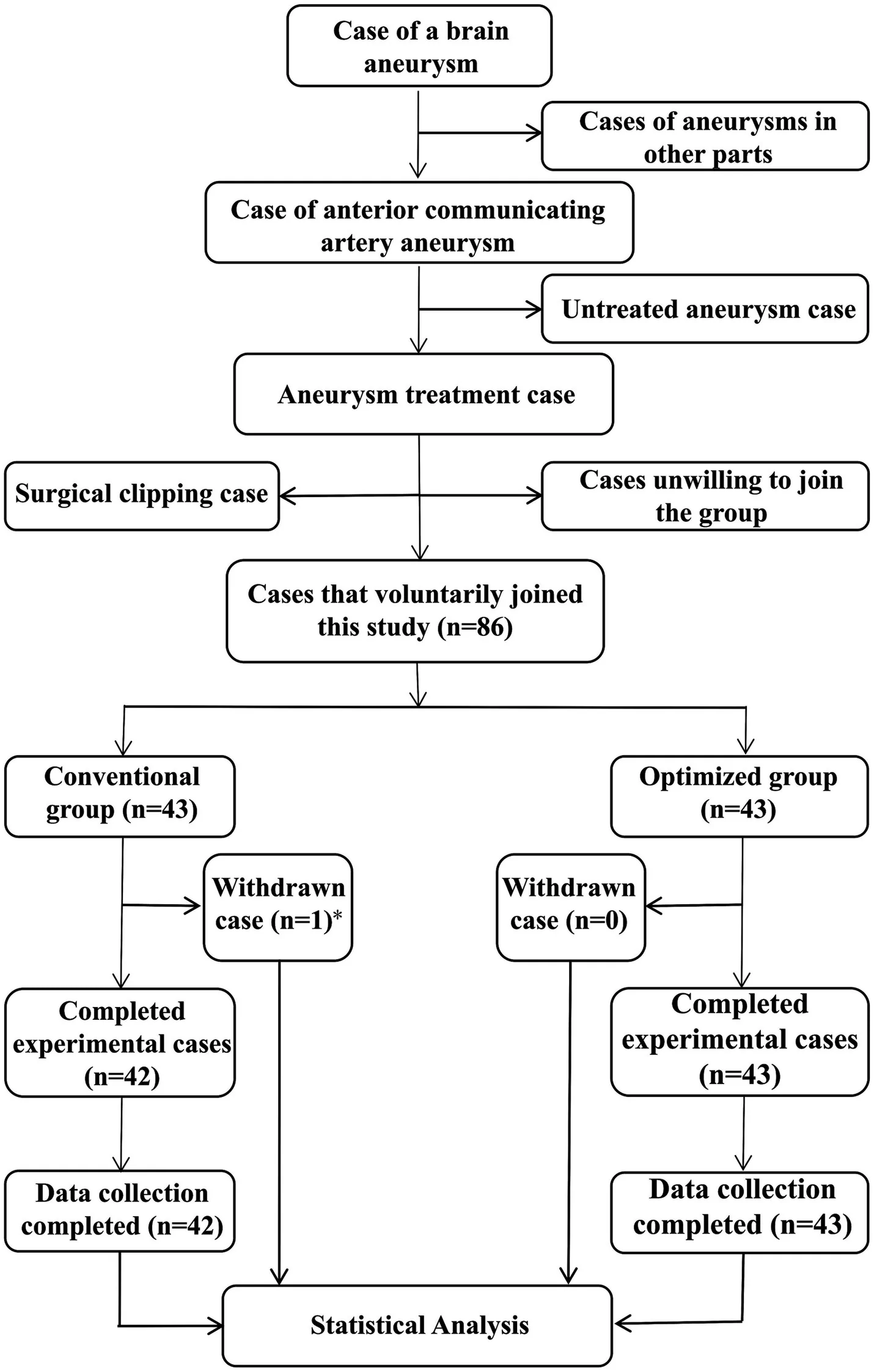
Flowchart of patient selection, group allocation, follow-up, and inclusion in the final statistical analysis. *One patient experienced aneurysm rupture before the procedure and developed unstable vital signs; the procedure was not performed, and participation in the trial was discontinued.

All patients with ruptured intracranial aneurysms (RIA) and unruptured intracranial aneurysms (UIA) were admitted to the Department of Cerebrovascular Diseases. Upon admission, the patient immediately enters the green channel for stroke, where a multidisciplinary team (including experts in neurology, neurosurgery, imaging, and critical care) conducts a comprehensive evaluation. Key indicators such as Glasgow Coma Scale (GCS), WFNS grading, Hunt Hess grading, NIHSS score, Modified Fisher Scale (mFS), and Modified Rankin Scale (mRS) are recorded, and personalized treatment plans are developed based on this. After fully informing the pros and cons of MC and EVT, patients and their families voluntarily choose EVT treatment and sign an informed consent form. Patients who meet the criteria will be randomly divided into the two groups. Except for the core variable of head position, the two groups of patients will maintain consistency in key clinical aspects such as anesthesia plan, selection of interventional surgical path, brands and models of embolization materials, and use of antiplatelet therapy, etc. The overall study workflow is shown in Figure 1.

### 2.3 Randomization and blinding

The study used a strict randomization and single-blind design. An independent research assistant, who was not involved in patient screening, procedures, or data collection, generated a computer-based randomization sequence and allocated patients in a 1:1 ratio to the conventional group or the optimized group. The allocation sequence was placed in sequentially numbered, opaque sealed envelopes. After preoperative preparation and immediately before transfer to the operating room, the corresponding envelope was opened by the operating surgeon in a preoperative consultation area to reveal group assignment. This process concealed allocation until assignment. Due to the nature of head positioning, blinding of the operating surgeon was not feasible. Therefore, a single-blind design was adopted, in which an independent neuro-interventional physician, who was not involved in the procedures, was responsible for intraoperative data recording and postoperative follow-up assessment. This evaluator remained blinded to group allocation throughout the study, thereby minimizing assessment bias.

### 2.4 Interventions

**Figure 2:**
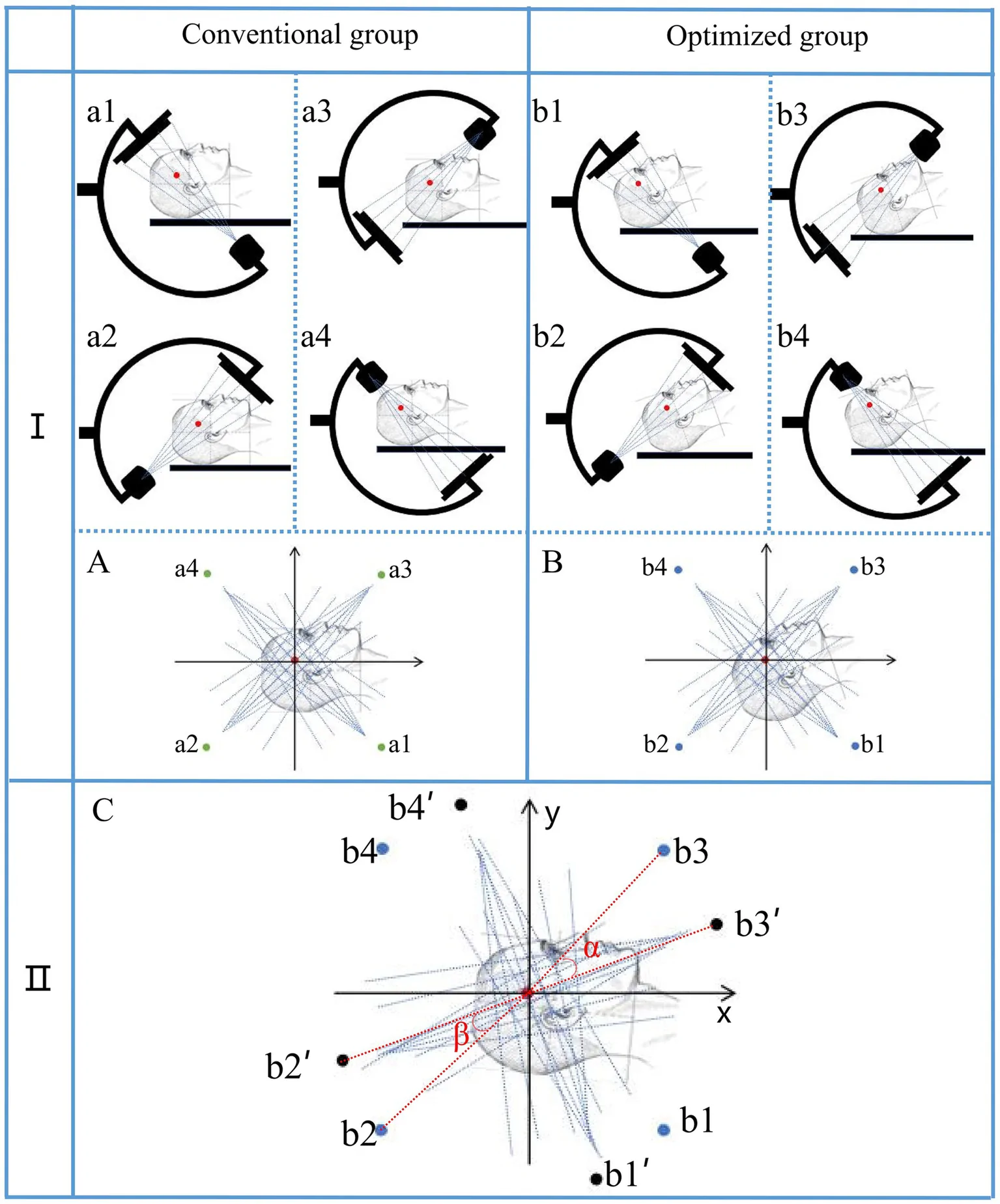
Comparison of the sagittal fluoroscopic field between the conventional and optimized groups. The red dot represents the ACoAA. Green dots indicate the four extreme C-arm positions in the conventional group, whereas blue and black dots indicate the simulated extreme C-arm positions before and after correction in the optimized group, respectively. a1–a4 and b1–b4 denote the four extreme C-arm positions for the conventional and optimized groups, respectively. Panels **A** and **B** show the simulated sagittal projection range and the four extreme C-arm positions in the conventional and optimized groups, respectively. Panel **C** shows the shift in the simulated extreme C-arm positions after correction (b1–b4 to b1′–b4′). The *α* and *β* angles represent the additional sagittal projection angles gained after head-position optimization.

The intervention was based on the premise that head positioning can alter the effective working range without changing the fixed mechanical limits of the C-arm. Due to inherent hardware limitations, the mechanical range of motion of the C-arm is fixed, and the maximal angulation of the X-ray tube is predetermined; therefore, the operative working space cannot be actively increased. However, head positioning can be adjusted to indirectly optimize and expand the effective imaging space, thereby improving the X-ray exposure range. A schematic illustration of the sagittal plane of the study model is shown in Figure 2.

**Figure 3:**
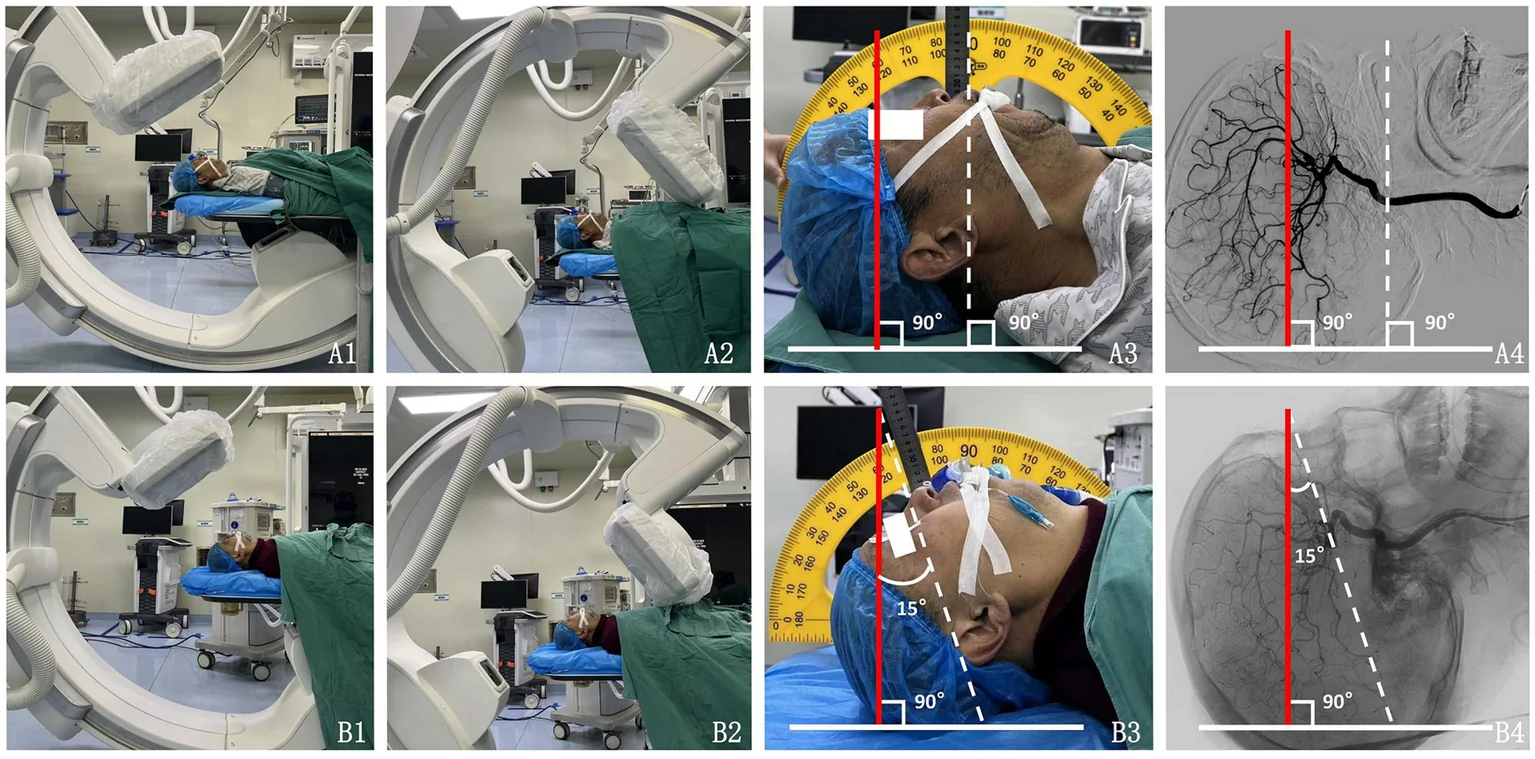
Sagittal views of patient positioning in the conventional and optimized groups. Panels A1 and A2 show the maximum Towne and reverse Towne angulations achievable with the C-arm, respectively, in the conventional group. Panel A3 shows a sagittal view of head positioning in the conventional group, with the semicircular protractor indicating an angle of 90°. Panel A4 shows the corresponding intraoperative sagittal angiographic image. Panels B1 and B2 show the maximum Towne and reverse Towne angulations achievable with the C-arm, respectively, in the optimized group. Panel B3 shows a sagittal view of head positioning in the optimized group, with the semicircular protractor indicating an angle of 105°. Panel B4 shows the corresponding intraoperative sagittal angiographic image. The solid white line represents the horizontal plane of the operating table, the solid red line represents the line perpendicular to the operating table, and the dashed white line represents the anteroposterior axis of the skull.

Figure 3 illustrates the two positioning strategies. In the conventional-position group, patients were placed supine with the head in a neutral midline position, and the cranial reference axis was positioned at 90° relative to the operating table. In the optimized-position group, the head was extended posteriorly by 15° from the conventional position, resulting in a cranial reference angle of 105°. Positioning was performed jointly by experienced anesthesiologists and nurses and verified using a level, protractor, and right-angle ruler. This standardized description of “15° posterior extension from 90° to 105°” is used throughout the manuscript.

### 2.5 Outcome measures

Intraoperative outcomes included the following: (1) total procedure time, defined as the interval from induction of anesthesia to completion of the procedure, removal of all vascular sheaths, and hemostatic compression at the puncture site; (2) stent-delivery catheter placement time, defined as the interval from successful entry of the stent-delivery catheter into the femoral arterial sheath to stable and accurate placement of the catheter tip in the distal parent artery; (3) embolization catheter placement time, defined as the interval from the initiation of microguidewire advancement through the embolization catheter to successful delivery of the catheter tip into the aneurysm sac with stable coil deployment; (4) coil embolization time, defined as the interval from stable microcatheter positioning within the aneurysm and initiation of the first-coil deployment to completion of coil embolization with dense aneurysm packing; (5) number of coils used, recorded as the total number of coils of all sizes and types deployed during the procedure; (6) sten-usage rate, defined as the proportion of patients who underwent adjunctive stent-assisted coiling; and (7) perioperative complication, including all adverse events such as vasospasm, parent- or branch-artery dissection, thromboembolism, perforator occlusion, and intraprocedural aneurysm rupture.

### 2.6 Statistical analysis

Statistical analyses were performed using SPSS version 26.0 software (IBM Corp., Armonk, NY, United States). Continuous variables conforming to a normal distribution were expressed as the mean ± standard deviation (SD), and comparisons between two groups were performed using the independent-samples *t-*test. Non-normally distributed continuous variables were summarized as the median (interquartile range, IQR) and compared using the Mann–Whitney *U* test for two-group comparisons. Categorical variables were summarized as frequencies and percentages and compared using the Chi-square test or Fisher’s exact test, as appropriate. A two-sided *p* < 0.05 was considered statistically significant.

## 3 Results

### 3.1 Baseline characteristics

A total of 86 patients were enrolled in this study, including 43 patients in the conventional group and 43 patients in the optimized group. In the conventional group, one patient experienced aneurysm rupture before the procedure and developed unstable vital signs; therefore, the procedure was not performed and the patient was withdrawn from the study. Ultimately, 85 patients were included in the final analysis: 42 in the conventional group and 43 in the optimized group.

The two groups were generally comparable with respect to demographic characteristics and baseline comorbidities. There were no statistically significant differences in age, sex distribution, or aneurysm status (RIA vs. UIA) between the conventional group and the optimized group patients. Among the baseline characteristics, BMI was slightly higher in the conventional group than in the optimized group (23.13 ± 2.78 vs. 22.04 ± 2.28 kg/m^2^, *p* = 0.048). Considering that the BMI difference was small and not strongly correlated with the core research indicators, it is believed that it does not affect the validity of the research conclusions. No statistically significant between-group differences were observed in the remaining baseline variables, including hypertension, admission blood pressure, diabetes, blood glucose, glycated hemoglobin, smoking history, and alcohol consumption (all *p* > 0.05) (Table 1).

**Table 1:** Baseline characteristics of the conventional and optimized position groups.

Characteristic	Conventional group (*n* = 42)	Optimized group (*n* = 43)	*p*-value
Age (years)	57.81 ± 10.08	55.91 ± 9.15	0.364
Sex [*n* (%)]			0.337
Male	23 (54.8%)	19 (44.2%)	
Female	19 (45.2%)	24 (55.8%)	
Aneurysm status [*n* (%)]			0.527
RIA	34 (81.0%)	37 (86.0%)	
UIA	8 (19.0%)	6 (14.0%)	
Body mass index (BMI) (kg/m^2^)	23.13 ± 2.78	22.04 ± 2.28	0.048
History of hypertension [*n* (%)]	12 (28.6%)	10 (23.3%)	0.577
Admission systolic blood pressure (mmHg)	146.40 ± 22.47	146.16 ± 22.41	0.960
Admission diastolic blood pressure (mmHg)	85.98 ± 14.32	90.88 ± 17.58	0.153
History of diabetes [*n* (%)]	3 (7.1%)	2 (4.7%)	0.676
Blood glucose (mmol/L)	7.08 ± 2.77	7.00 ± 1.82	0.877
Glycated hemoglobin (%)	5.95 ± 0.93	5.72 ± 0.41	0.129
Smoking history [*n* (%)]	13 (31.0%)	16 (37.2%)	0.541
Alcohol consumption [*n* (%)]	13 (31.0%)	16 (37.2%)	0.541

BMI, body mass index; RIA, ruptured intracranial aneurysm; UIA, unruptured intracranial aneurysm.

### 3.2 Comparative analysis of procedural time and treatment characteristics

As shown in Table 2, compared with the conventional group, the optimized group demonstrated significantly shorter total procedure time for both SACE and SCE (SACE: 124.74 ± 45.11 min vs. 166.96 ± 38.77 min, *p* = 0.001; SCE: 88.33 ± 22.77 min vs. 143.50 ± 32.76 min, *p* < 0.001). No significant difference was observed in procedure preparation time between the two groups (12.42 ± 7.54 min vs. 13.07 ± 6.20 min, *p* = 0.668). The optimized group showed significantly reduced stent-delivery catheter placement time (31.90 ± 14.35 min vs. 49.17 ± 16.79 min, *p* < 0.001) and embolization catheter placement time (23.30 ± 8.98 min vs. 44.76 ± 15.14 min, *p* < 0.001). In terms of coil embolization time, the optimized group achieved shorter durations in both SACE (31.26 ± 11.02 min vs. 43.68 ± 17.41 min, *p* = 0.006) and SCE procedures (27.04 ± 9.07 min vs. 51.93 ± 22.55 min, *p* < 0.001).

**Table 2:** Comparison of procedural times and treatment characteristics between the conventional group and optimized groups.

Procedural variable	Conventional group (*n* = 42)	Optimized group (*n* = 43)	*p*-value
Total procedure time			
SACE	166.96 ± 38.77	124.74 ± 45.11	0.001
SCE	143.50 ± 32.76	88.33 ± 22.77	<0.001
Procedure preparation time (min)	13.07 ± 6.20	12.42 ± 7.54	0.668
Stent-delivery catheter placement time (min)^*^	49.17 ± 16.79	31.90 ± 14.35	<0.001
Embolization catheter placement time (min)	44.76 ± 15.14	23.30 ± 8.98	<0.001
Coil embolization time			
SACE	43.68 ± 17.41	31.26 ± 11.02	0.006
SCE	51.93 ± 22.55	27.04 ± 9.07	<0.001
Number of coils used	3.93 ± 1.50	3.91 ± 1.23	0.707
Stent use [*n* (%)]	28 (66.7%)	19 (44.2%)	0.037

^*^In some cases, simple coil embolization can be clearly determined preoperatively, and stent microcatheters will not be pre-implanted during the operation for such patients; in all other cases, stent microcatheters are pre-implanted during the operation. Therefore, this figure should be greater than or equal to the number of patients using stents, and less than the total number of patients. SACE, stent-assisted coil embolization; SCE, standalone coil embolization.

The results in Table 2 also showed that the optimized group had a significant decrease in stent utilization rate, with the conventional group having a stent utilization rate of 66.7%, while the optimized group had a stent utilization rate of only 44.2% (*p* = 0.037). Although the optimized group used fewer stents, there was no statistically significant difference in the number of embolization coils used between the two groups (3.93 ± 1.5 vs. 3.91 ± 1.23, *p* = 0.707).

### 3.3 Comparison of the improvement in neurological function prognosis

Compared with the conventional group, the optimized group had a higher postoperative GCS score (14.81 ± 1.73 vs. 13.10 ± 3.69, *p* = 0.004) and a greater change in GCS score (0.83 ± 1.99 vs. −0.60 ± 2.98, *p* = 0.009). Although postoperative NIHSS and modified Fisher Scale scores were numerically lower in the optimized group (NIHSS: 1.21 ± 4.54 vs. 3.79 ± 8.89, *p* = 0.095; modified Fisher Scale: 0.09 ± 0.45 vs. 0.19 ± 0.75, *p* = 0.460), these between-group differences were not statistically significant. However, the changes in NIHSS (3.28 ± 6.67 vs. 2.14 ± 8.29, *p* = 0.027) and modified Fisher Scale scores (2.02 ± 1.09 vs. 1.76 ± 1.29, *p* = 0.010) were greater in the optimized group.

Postoperative Barthel Index and Katz Index were higher in the optimized group than in the conventional group (Barthel Index: 95.35 ± 15.80 vs. 84.52 ± 30.16, *p* = 0.042; Katz Index: 11.67 ± 1.89 vs. 10.17 ± 3.45, *p* = 0.016). The postoperative FIM score was numerically higher in the optimized group, but the difference was not statistically significant (122.42 ± 18.92 vs. 115.12 ± 25.71, *p* = 0.140). The optimized group also showed greater improvements in the Barthel Index (18.26 ± 22.38 vs. 10.71 ± 24.62, *p* < 0.001), Katz Index (2.58 ± 2.49 vs. 1.24 ± 2.67, *p* < 0.001), and FIM score (12.14 ± 19.89 vs. 7.14 ± 19.72, *p* = 0.001).

Neither the postoperative mRS score nor the change in mRS differed significantly between the groups (postoperative: 1.35 ± 1.22 vs. 1.79 ± 1.34, *p* = 0.147; change: −0.21 ± 1.61 vs. −0.55 ± 1.78, *p* = 0.132) (Table 3).

**Table 3:** Comparison of the improvement in neurological function prognosis.

Variable	Scoring period	Conventional group (*n* = 42)	Optimized group (*n* = 43)	*p*-value
GCS	Preoperative	13.69 ± 2.11	13.98 ± 1.87	0.414
	Postoperative	13.10 ± 3.69	14.81 ± 1.73	0.004
	Change	−0.6 ± 2.98	0.83 ± 1.99	0.009
NIHSS Score	Preoperative	5.93 ± 7.14	4.49 ± 6.72	0.278
	Postoperative	3.79 ± 8.89	1.21 ± 4.54	0.095
	Change	2.14 ± 8.29	3.28 ± 6.67	0.027
Modified Fisher Scale Score	Preoperative	1.95 ± 1.20	2.12 ± 1.08	0.372
	Postoperative	0.19 ± 0.75	0.09 ± 0.45	0.460
	Change	1.76 ± 1.29	2.02 ± 1.09	0.01
Barthel Index	Preoperative	73.81 ± 25.59	77.09 ± 23.40	0.511
	Postoperative	84.52 ± 30.16	95.35 ± 15.80	0.042
	Change	10.71 ± 24.62	18.26 ± 22.38	<0.001
KatzI Index	Preoperative	8.93 ± 2.33	9.09 ± 2.66	0.702
	Postoperative	10.17 ± 3.45	11.67 ± 1.89	<0.001
	Change	1.24 ± 2.67	2.58 ± 2.49	0.016
Total score of functional independence measure (FIM)	Preoperative	107.98 ± 20.35	110.28 ± 22.11	0.608
	Postoperative	115.12 ± 25.71	122.42 ± 18.92	0.140
	Change	7.14 ± 19.72	12.14 ± 19.89	0.001
Modified Rankin Scale (mRS)	Preoperative	1.24 ± 1.88	1.14 ± 1.65	0.768
	Postoperative	1.79 ± 1.34	1.35 ± 1.22	0.147
	Change	-0.55 ± 1.78	−0.21 ± 1.61	0.132

GCS, Glasgow Coma Scale; NIHSS, National Institutes of Health Stroke Scale.

### 3.4 Incidence of perioperative complications

As shown in Table 4, the overall incidence of perioperative complications was lower in the optimized group than in the conventional group, but the difference was not statistically significant (6.98% vs. 11.90%, *p* = 0.483). Specifically, intraprocedural aneurysm rupture occurred in 2 patients in the conventional group (4.76%), whereas no such event was observed in the optimized group (*p* = 0.496). Thromboembolic events were also less frequent in the optimized group than in the conventional group, but the between-group difference was not statistically significant (2.33% vs. 4.76%, *p* = 0.616). Vasospasm occurred in 1 patient in the conventional group (2.38%) and in 2 patients in the optimized group (4.65%), with no significant difference observed (*p* = 1.000). No femoral artery access-site hematoma was reported in either group.

**Table 4:** Comparison of perioperative complications between the two groups.

Complications	Conventional group (*n* = 42)	Optimized group (*n* = 43)	*p*-value
Intraprocedural aneurysm rupture [*n* (%)]	2 (4.76%)	0 (0%)	0.496
Thromboembolic event [*n* (%)]	2 (4.76%)	1 (2.33%)	0.616
Vasospasm event [*n* (%)]	1 (2.38%)	2 (4.65%)	1.000
Femoral access-site hematoma [*n* (%)]	0 (0%)	0 (0%)	—
Other complications [*n* (%)]	0 (0%)	0 (0%)	—
Overall complications [*n* (%)]	5 (11.90%)	3 (6.98%)	0.483

Overall, these findings suggest that the optimized strategy may be associated with a lower overall complication rate, particularly with respect to intraprocedural aneurysm rupture and thromboembolic events; These findings should be interpreted descriptively because none of the between-group differences in individual or overall complications reached statistical significance, possibly due to the limited sample size.

## 4 Discussion

As a type of IA with high incidence rate and significant risk of rupture, ACoAA treatment has always been an important topic in the field of neurology (Gundersen et al., 2025). With ongoing technical advances, EVT has become a principal treatment option for ACoAA (Luckrajh et al., 2022; Sattari et al., 2023). The continuous progress of EVT highlights the importance of fully mastering the anatomical knowledge of ACoAA complex (Kayembe et al., 1984; Clarke et al., 2023). ACoAA involves complex vascular networks and important perforating arteries, and how to fully expose lesions and protect surrounding structures during surgery remains a clinical challenge (Ozdemir et al., 2014). As one of the key factors in surgical operations, the impact of head position on surgical field of view, instrument placement, and treatment efficacy has not been fully studied (Chen et al., 2020; Lee et al., 2025).

The traditional position is usually a parallel relationship between the anterior posterior line of the patient’s head and the vertical line of the bed surface (i.e., the center of the head and neck, perpendicular to the bed). During the operation, the surgeon manually adjusts the multi angle projection of the C-arm (such as Towne projection) for anatomical positioning. However, the ACoA complex region has a complex structure and high variability rate (Staarmann et al., 2017; Feng et al., 2025). A meta-analysis identified 42 types of variations in the anatomical structure of the ACoA complex (Kashtiara et al., 2024). Saha et al. (2024) pointed out that ACoA has an overall variation rate of 40–50%. The complexity and high variability of local vascular anatomy ultimately lead to a significant decrease in the clarity and spatial perception of surgical field exposure. In the traditional AP position, the vertical movement space of the C-arm is limited. Although 3D Roadmap technology can be used to plan multi angle treatment paths, in actual surgical operations, many theoretical optimal angles are difficult to fully achieve within the adjustable range of physical equipment (Dowlati et al., 2020).

In the optimized group, the head space for X-ray irradiation has been expanded to a certain extent. In both the conventional group and the optimized group, although the stroke of the C-arm head is limited by hardware, the optimized group actively adjusted the head position (head optimized position), which allowed the head to be illuminated in three-dimensional space to a certain extent, expanding the spatial range of *α* angle and *β* angle in the sagittal plane. These two additional spatial angles can provide better exposure angles for ACoAA in the anterior (toward the frontal lobe), posterior (toward the optic chiasm), upward (toward the hypothalamus), and downward (toward the skull base) directions, with better exposure angles for ACoAA in the neck tangent and radial directions. Wiśniewski et al. (2026) believe that the orientation of ACoAA is a key factor in determining the flow mechanics, which will increase the risk of ACoAA rupture. Forward growing aneurysms are the anterior communicating aneurysms with the highest incidence rate and are more prone to rupture. In our study, the proportion of anterior aneurysms in the conventional group and the optimized group was 42.86 and 51.16%, respectively (there was no statistical difference between the two groups, as shown in Table 3). Our sample data is highly consistent with Wiśniewski’s research findings, which further support the validity of our research results.

In this study, the optimized posture refers to replacing the traditional vertical posture with a tilt angle of 15° (i.e., 105°) for the head, instead of the previous 90°vertical posture, which fully utilizes the value of Towne projection in imaging diagnosis(Mori et al., 2022), taking into account factors such as anatomy, equipment, and mechanics. In the past, Mori also confirmed through retrospective analysis that the Towne position is significantly better than the conventional anterior posterior position in diagnosing intracranial small vessel lesions (Zhang et al., 2019). On the other hand, a 15° backward tilt optimized head position can fine tune the natural curvature and relative position between ACoA, aneurysms, and their associated blood vessels, making it easier for the catheter to approach the tumor along the direction of blood flow; At the same time, this reclining posture can alleviate the geometric distortion in the projection of the C-arm to a certain extent, improve the accuracy of 3D reconstruction, and enable the operating physician to have a more intuitive understanding of the 3D space construction of the aneurysm neck and the parent artery during surgery.

The results of this study showed that the optimized group made significant progress in surgical efficiency compared to the conventional group, particularly in reducing key time consuming indicators such as total surgical duration, placement time of stent and embolization catheters, and embolization operation time, demonstrating the practical value of head position optimization in improving surgical fluency and reducing the need for repetitive procedures. Another noteworthy finding is that the optimized group had significantly lower stent usage rates than the conventional group (44.2% vs. 66.7%), while there was no significant difference in the number of embolization coils used between the two groups. This result indicates that optimizing the position helps to reduce reliance on auxiliary stents while ensuring the effectiveness of embolization. Although stents can provide good aneurysm neck coverage and blood flow remodeling in ACoAA intervention therapy, their use also comes with additional operational steps and may increase surgical complexity and higher risk of complications (Zhang et al., 2019). By optimizing the position, it is easier for the catheter to achieve dense embolization within the tumor without stent assistance, thus omitting the use of stents in some cases. This not only simplifies the surgical process, but also may reduce the long-term medication burden and re intervention risk of patients after surgery, and has certain health and economic value.

Several short-term neurological and functional measures favored the optimized-position group. Postoperative GCS scores and the changes in GCS, NIHSS, and modified Fisher Scale scores were significantly more favorable in the optimized group. In contrast, postoperative NIHSS and modified Fisher Scale scores were numerically lower, but the between-group differences were not statistically significant. Postoperative Barthel Index and Katz Index scores and the changes in Barthel Index, Katz Index, and FIM scores were significantly higher in the optimized group; the postoperative FIM score was numerically higher but did not differ significantly. In contrast, neither postoperative mRS scores nor changes in mRS differed significantly between the groups. The overall complication rate was numerically lower in the optimized-position group, but neither the overall rate nor any individual complication differed significantly. The safety findings should therefore be interpreted cautiously and primarily support the absence of an evident increase in perioperative complications.

Several limitations should be acknowledged. First, this was a single-center study with a relatively small sample, which limited the statistical power to evaluate uncommon complications. Second, follow-up focused on short-term outcomes; long-term neurological recovery, aneurysm occlusion, recurrence, and retreatment were not assessed. Third, the operating surgeon could not be blinded, and procedural performance may have been influenced by operator experience despite blinded outcome assessment. Fourth, a fixed 15° angle may not be optimal for every anatomical configuration or angiography system. Multicenter studies with larger samples, longer follow-up, and anatomy-specific positioning analyses are warranted.

## 5 Conclusion

In the endovascular treatment of anterior communicating artery aneurysms, optimizing the head position can effectively improve surgical efficiency, reduce the use of stents, without increasing surgical risks, and help improve patients’ short-term neurological function after surgery. This measure is simple and easy to implement, with good clinical promotion value and application prospects.

## Data Availability

The raw data supporting the conclusions of this article will be made available by the authors, without undue reservation.
